# Early Prediction of Corpus Luteum Functionality Using an Imaging Software

**DOI:** 10.3389/fvets.2020.00299

**Published:** 2020-06-18

**Authors:** Angela Salzano, Marco Russo, Giuseppe Anglani, Francesca Licitra, Gianluigi Zullo, Alessio Cotticelli, Gerardo Fatone, Giuseppe Campanile

**Affiliations:** ^1^Department of Veterinary Medicine and Animal Production, University of Naples Federico II, Naples, Italy; ^2^Istituto Zooprofilattico Sperimentale della Sicilia, Palermo, Italy

**Keywords:** corpus luteum, buffaloes, blood flow area, progesterone, pregnancy diagnosis

## Abstract

The present study aimed to assess the applicability of luteal blood flow data acquired through the use of color Doppler ultrasonography and a post-processing analysis tool (ImageJ) for predicting pregnancy in buffaloes (*Bubalus bubalis*). The experiment was carried out on 59 multiparous Italian Mediterranean buffaloes that underwent synchronization of estrus and fixed-time artificial insemination (TAI). Corpus luteum features (size: CLS and blood flow: BFA) were taken from Day 5 to 10 after TAI and retrospectively measured with ImageJ. In the same period, blood samples were taken to assess progesterone (P_4_) concentrations. Pregnancy diagnosis was carried out on Day 45 by ultrasound and confirmed on Day 70 post-TAI. Differences in CLS, BFA, and P_4_ concentrations from Day 5 to 10 after TAI measured between groups were analyzed by ANOVA repeated measures as were differences within each day of measuring. Buffaloes that established a pregnancy (*n* = 29; 55%) had larger CLS (2.2 ± 0.1 vs. 1.9 ± 0.1 cm^2^; *P* < 0.01), higher BFA (0.6 ± 0.0 vs. 0.4 ± 0.0 cm^2^; *P* < 0.01), and higher P_4_ blood level (1.8 ± 0.1 vs. 1.4 ± 0.1; *P* < 0.01) during Day 5–10 as compared to not-pregnant buffaloes (*n* = 22). Throughout the entire period, the first feature that changed between groups was P_4_ blood concentration at Day 7 (1.7 ± 0.1 vs. 1.2 ± 0.1; *P* < 0.05) followed by BFA at Day 8 (0.6 ± 0.0 vs. 0.5 ± 0.0; *P* < 0.05), respectively, in pregnant and not-pregnant animals. The ROC analyses indicated that P_4_ was able to predict pregnancy since Day 5 (*P* < 0.05) although a more reliable result could be obtained from Day 8 (*P* < 0.01). At Day 10, it was possible to set a cutoff value for every parameter taken into account. The logistic regression analysis showed that pregnancy was positively influenced by P_4_ concentration (odds ratio 534.127; *P* < 0.01) and BFA (odds ratio 744.893; *P* < 0.01). In conclusion, the use of color Doppler ultrasonography, together with ImageJ, identified different patterns of BFA between pregnant and not-pregnant buffaloes starting from Day 8 post-TAI.

## Introduction

The corpus luteum (CL) is essential for establishing and maintaining pregnancy in females (1–3). The advent of color Doppler ultrasound imaging made it possible to examine the intensity of CL blood flow (4) and allows for the generation of real-time information about CL functionality in different phases of the estrous cycle. It is known that angiogenesis is positively correlated with blood P_4_ concentrations during CL development and that the apoptosis of endothelial cells is involved in both functional and structural luteolysis (5). Regarding important features of luteal blood flow, a study demonstrated that the blood flow area (BFA) measured on Day 7 and paired BFA and time average maximum velocity (TAMV) recorded on Day 14 could predict pregnancy in cows (6). Indeed, the most important application for the assessment of CL blood flow is the early detection of not-pregnant females (7–9). A previous study showed, in buffaloes, a strong correlation between CL vascularization and blood flow and P_4_ synthesis and release (10, 11). It has also been demonstrated that buffaloes with a greater CL blood flow on Days 10, 20, and 25 after timed artificial insemination (TAI) are more likely to become pregnant, probably due to increased angiogenesis (12). Moreover, Neglia et al. (7) focused on the period between Day 5 and 10 post-AI and revealed that a higher size and vascularization of the CL, together with higher P_4_ concentrations in that specific time window, are correlated with pregnancy. In particular, buffaloes with higher CL vascularization on Day 8 after TAI had a higher pregnancy rate, and there is a positive relationship between TAMV values, P_4_ concentrations, and pregnancy rates.

The color Doppler ultrasound imaging approach has the potential to improve buffalo reproductive efficiency by allowing for earlier resynchronization of open females and, consequently, a reduction in the interval between services (5, 13). Moreover, the use of a free post-processing tool such as ImageJ could be of great advantage for management strategies that use intensive resynchronization schedules to improve reproductive performance.

Therefore, the present study aims to evaluate the applicability of BFA obtained by using color Doppler ultrasonography and post-processing analyses for predicting pregnancy. Specifically, we addressed questions regarding: (1) changes in CL size (CLS), BFA, and P_4_ blood levels from Day 5 to 10 after TAI; (2) how early changes in vascularization, morphology (size), and function of the CL are different in prospective pregnant and not-pregnant buffaloes; and (3) whether vascular image analysis can predict conception using ROC analysis, logistic, and linear regression models.

## Materials and Methods

The Ethical Animal Care and Use Committee of the Federico II University of Naples approved the experimental design and animal treatments. Written informed consent was obtained from the owners for the participation of their animals in this study.

### Animals

The experiment was carried out on 59 multiparous Italian Mediterranean buffalo cows at 133 ± 12 days in milk (DIM) during the breeding season (October–November 2018). Animals involved in the trial were selected from a larger group of buffaloes by a clinical examination that included (1) rectal palpation of the ovaries for follicular development, (2) a functional CL, and (3) no abnormalities of the reproductive tract. The animals were maintained in open yards that allowed 15 m^2^ per buffalo and a manger space of 80 cm. The buffaloes were fed a total mixed ration consisting of 55% forage and 45% concentrate, containing 0.91 milk forage units/kg of dry matter and 15% crude protein. Moreover, the body condition score (BCS) of each buffalo was recorded weekly using a 1–9 scale (14).

### Estrus Synchronization and AI

Ovsynch protocol with fixed-time AI (TAI) was used to synchronize the animals. This protocol was initially developed in cattle (15) and previously used in buffaloes (16). Briefly, a GnRH agonist (buserelin acetate, 12 mg; Receptal, Intervet) was administered on Day 0, a PGF2α analog (Luprostiol, 15 mg; Prosolvin, Intervet) was administered on Day 7, and a GnRH agonist (12 mg) was administered again on Day 9. Artificial inseminations were performed by the same technician 20 h after the second injection of GnRH. Because of the relatively low estrous behavior in buffaloes (17), animals underwent rectal palpation (immediately before TAI), and only buffaloes considered to be in estrus were inseminated.

### Corpus Luteum Development and Blood Flow

**Figure 1:**
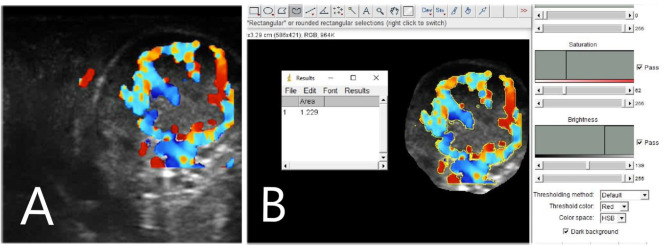
Corpus luteum (CL) blood flow assessment with ImageJ post-processing software. In **(A)** a CL blood flow picture, made with color Doppler mode and in **(B)** the same picture during the post-processing analysis with the software.

Corpus luteum ultrasonography examinations were performed daily from Day 5 to 10 post-TAI by using a portable MyLab Goldvet 30 ultrasound unit (Esaote, Italy) equipped with a 7.5 MHz linear transducer adapted for transrectal examination in large animals. Corpus luteum features (CLS and BFA) were examined daily. To obtain a better definition of the CL, once the ovary was visualized, the image was adjusted and then frozen to measure both the long and short axes, and the color Doppler mode was activated. Color gain setting, velocity setting, and a color-flow filter setting were standardized during all the procedures, and all the analyses were carried out by the same technician. Real-time B-mode/color Doppler images of the continuous scans of the CL were recorded and then analyzed retrospectively. B-mode and color flow mode short video clips (7 s duration) were stored in the internal memory of the ultrasound machine. Later, as described by Siqueira et al. (9), the CL area and the area of the cavity (if present) were calculated using the machine's internal calipers. To justify fluid-filled cavities, CLS was assessed by subtracting the area of the cavity from the entire CL area. To determine the blood flow area, a color Doppler image at the maximum diameter of the CL was examined using an open-source post-processing image tool, such as ImageJ (version 1.49; National Institutes of Health, Bethesda, MD) (18). To assess the area of colored pixels within the CL, an indirect estimation of CL blood flow area (BFA) was made. In particular, the BFA of the corpus luteum was calculated by analyzing the eco-color Doppler images, selecting the colored areas (not including artifacts), and setting the scale (pixels numbers = 1 cm) in each image using the references on the side of the eco-color Doppler images (Figure 1).

### Progesterone

Starting from Day 5 to 10 after TAI, blood levels of P_4_ were assessed by RIA (19, 20) to determine CL functionality. Blood samples from the jugular vein were collected into heparinized tubes on the same days as the ultrasound examinations. The samples were then centrifuged at 800 × g for 15 min and the serum was stored at −20°C until analysis. The minimum detectable amount of P_4_ was 2.0 ± 0.09 pg/ml, and the intra-assay and inter-assay coefficients of variation were 6.1 and 11.9%, respectively. Values of 1.2 ng/ml were considered indicative of the presence of a functional CL, according to previous studies (7).

### Embryonic Development and Pregnancy

Pregnancy diagnosis was carried out on Day 45 post-TAI by using the same ultrasound machine described above. Pregnancy status was confirmed on Day 70 post-TAI. Animals that were pregnant on Day 45 and not pregnant on Day 70 were considered to have undergone fetal mortality.

### Statistical Analysis

All data were presented as the mean ± standard error, and all statistical analyses were performed using SPSS IBM version 22.0 statistical software (21). Differences in DIM and BCS between pregnant and not pregnant were analyzed by the Student *t*-test. Differences in BFA, CLS, and P_4_ concentrations from Day 5 to 10 after TAI measured between groups were analyzed by an ANOVA repeated-measures test as were differences within each day of measuring. Statistical significance was determined based on a *P*-value of 0.05. Changes (Δ) in dimensions of CLS, BFA, and P_4_ were assessed by subtracting the value of one observation from that of the previous ones: Period 1 (P1, Day 5–6), Period 2 (P2, Day 6–7), Period 3 (P3, Day 7–8), Period 4 (P4, Day 8–9), Period 5 (P5, Day 9–10), and Period 6 (P6, Day 5–10).

Stepwise forward linear regression was performed using the mean of BFA, CLS, P_4_, DIM, and BCS, including the differentials (Δ) for CLS, BFA, and P_4_, as independent variables to assess their relationships with CLS, BFA, and P_4_ in three different groups (all animals A, pregnant P, not pregnant NP). Logistic regression for pregnancy outcome was calculated using CLS, BFA, and P_4_ concentrations, DIM, and BCS as independent variables to evaluate whether one of the variables could be used to predict pregnancy. Furthermore, receiver operating characteristics (ROC) analyses were assessed, focusing on three features (CLS, BFA, and P_4_ concentrations) on Day 5–10 post-TAI to identify the optimal cutoff value for predicting pregnancy. The latter was determined from the data point minimizing the distance, reporting the relative sensitivity and specificity percentages and area under the curve (AUC) value.

## Results

### Pregnancy and CL Development and Function

Fifty-nine buffaloes were subjected to synchronization, but only 49 (83%) out of 59 responded to the synchronizing protocol and underwent TAI. On Day 45 and confirmed on Day 70, 27 out of 49 (55%) animals were pregnant. Both DIM and BCS did not differ between groups (134.2 ± 8.4 vs. 127.0 ± 7.8 days and 7.6 ± 0.4 vs. 7.4 ± 0.3, respectively, in pregnant and not-pregnant animals). The average corpus luteum area was similar from Day 5 to 10 in pregnant animals compared to their not-pregnant counterparts (Table 1) although a (*P* < 0.05) difference was recorded on Day 5. The first parameter that became different between groups was P_4_ at Day 7 (*P* < 0.05), followed by BFA at Day 8 (*P* < 0.05). At Day 10, both CLS and BFA were different (*P* < 0.01) between pregnant and not-pregnant buffaloes (Table 1). In terms of differences per each day within the two groups (P and NP), the BFA and the P_4_ concentration increased in a time-dependent manner in P group, and in the NP group, the same features started to decrease from Day 9 (Table 1).

**Table 1:** Differences in corpus luteum size (CLS), blood flow area (BFA), and P_4_ circulating concentrations between pregnant and non-pregnant buffaloes, during period from day 5 to 10 post-TAI.

**Day**	**CLS (cm**^****2****^**)**		**BFA (cm**^****2****^**)**		**P4 (pg/ml)**	
	**P (27)**	**NP (22)**	**P (27)**	**NP (22)**	**P (27)**	**NP (22)**
5	2.1 ± 0.3^a^	1.6 ± 0.1^b^	0.3 ± 0.0	0.3 ± 0.0	1.2 ± 0.1	1.1 ± 0.1
6	1.9 ± 0.1	1.8 ± 0.1	0.4 ± 0.0	0.4 ± 0.0	1.5 ± 0.1	1.3 ± 0.1
7	2.2 ± 0.1	1.9 ± 0.1	0.5 ± 0.0	0.4 ± 0.0	1.7 ± 0.1^a^	1.3 ± 0.1^b^
8	2.3 ± 0.1	2.0 ± 0.1	0.6 ± 0.0^a^	0.5 ± 0.0^b^	2.0 ± 0.1^A^	1.4 ± 0.1^B^
9	2.5 ± 0.1	2.2 ± 0.1	0.7 ± 0.0^A^	0.5 ± 0.0^B^	2.2 ± 0.1^A^	1.5 ± 0.1^B^
10	2.4 ± 0.1	2.0 ± 0.1	0.8 ± 0.0^A^	0.4 ± 0.0^B^	2.3 ± 0.1^A^	1.4 ± 0.1^B^

*Values are expressed as mean ± standard error*.

a,b *Values with different superscripts within adjacent rows are different; P < 0.05*.

A,B *Values with different superscripts within adjacent rows are different; P < 0.01*.

The average values from Day 5 to 10 post-TAI were greater (*P* < 0.01) in the P group than in the NP group (Table 2). In particular, the BFA was 1.5 times higher in the P group than in the NP group, and this was the higher rate compared to CLS and P_4_ concentration, which were, respectively, 1.16 and 1.29 times higher in the P group than in the NP group (Table 2).

**Table 2:** Differences in corpus luteum size (CLS), blood flow area (BLF), and P4 circulating concentrations mean values from day 5 to 10 post-TAI between pregnant (P; *n* = 27) and not pregnant (NP; *n* = 22) buffaloes.

**Group**	**n**	**CLS (cm^**2**^)**	**BFA (cm^**2**^)**	**P4 (pg/ml)**
P	27	2.2 ± 0.1^A^	0.6 ± 0.0^A^	1.8 ± 0.1^A^
NP	22	1.9 ± 0.1^B^	0.4 ± 0.0^B^	1.4 ± 0.0^B^

*Values are expressed as mean ± standard error*.

A,B *Values with different superscripts within adjacent rows are different; P < 0.01*.

### Prediction of Pregnancy

The ROC analyses indicated that P_4_ was the only predictor of pregnancy, among the others, that was able to detect pregnancy since Day 5 (Table 3). Based on the distance from the ideal point, our analysis defined the best P_4_ cutoff value as 0.91 pg/ml for the pregnancy prediction on Day 5 (sensitivity 77.8% and specificity 40.9%; *P* < 0.05). On Day 10 after TAI, the P_4_ cutoff value was set at 1.82 pg/ml with an increase in sensitivity (85.2%) and specificity (81.8%; *P* < 0.01). Regarding the other parameters, it was possible to set a cutoff value (0.59 cm^2^) for BFA only from Day 9 with a sensitivity of 70.4% and a specificity of 59.1% (*P* < 0.01). At Day 10, it was possible to set a cutoff value for every parameter taken into account (Table 3).

**Table 3:** Summary of the ROC analyses of the three (progesterone, P_4_; Corpus luteum area, CLS; Corpus luteum blood flow, BFA) independent variables from day 5 to 10 post-TAI.

**Day**	**Items**	**Cutoff value**	**AUC value**	**Sensitivity (%)**	**Specificity (%)**	***P*-value**
5	P_4_	0.94 pg/ml	0.71 ± 0.08	81.5	45.5	*P <* 0.05
7	P_4_	1.22 pg/ml	0.70 ± 0.07	77.8	40.9	*P <* 0.05
8	P_4_	1.43 pg/ml	0.83 ± 0.06	88.9	54.5	*P <* 0.01
9	P_4_	1.82 pg/ml	0.80 ± 0.07	81.5	68.2	*P <* 0.01
9	BFA	0.59 cm^2^	0.74 ± 0.07	70.4	59.1	*P <* 0.01
10	P_4_	1.82 pg/ml	0.91 ± 0.04	85.2	81.8	*P <* 0.01
10	BFA	0.63 cm^2^	0.94 ± 0.03	88.9	86.4	*P <* 0.01
10	CL	2.22 cm^2^	0.70 ± 0.08	70.4	63.6	*P <* 0.05

### Regression Analyses

Linear regression analyses of the parameters measured from Day 5 to 10 showed a relationship (*R*^2^ = 0.571; *P* < 0.01) only in not-pregnant animals between BFA, Δ5 P4 (D10–D9) and Δ3 P4 (D8–D7) as shown in the equation

$$\begin{array}{l} BFA\left(cm^{2}\right)=0.456+0.196\Delta 5P4\left(D10-D9\right)-0.131\\ \;\Delta 3P4\left(D8-D7\right). \end{array}$$

The logistic regression analysis showed that pregnancy outcome prediction, from Day 5 to 10 after TAI, was positively influenced by P_4_ concentration (odds ratio 534.127; *P* < 0.01) and BFA (odds ratio 744.893; *P* < 0.01). None of the other independent variables (CLS, DIM, BCS) analyzed showed relationships with pregnancy.

## Discussion

In this study, we demonstrated that a post-processing tool, such as ImageJ, used to analyze Doppler images could be a suitable tool for studying CL blood flow, whereas the common examinations of the CL area did not seem to be as useful for that purpose. The use of post-processing tools to analyze images was proposed as an alternative to optimize the interpretation of sonographic images of the CL (22, 23) and follicular wall (24, 25). In our study, pregnant buffaloes showed, at Day 10 post-AI, higher BFA and P_4_ blood concentration (*P* < 0.01) but not higher CLS compared to their not-pregnant counterparts. This latter finding is in agreement with another study (13), in which no differences in the CL area were recorded during this period (Day 5–10) between pregnant and not-pregnant buffaloes. It is already known that, since Day 5 post-TAI, buffaloes that showed lower CL vascularization had a lower pregnancy rate (7). Indeed, an appropriate BFA is necessary for the release of P_4_ into the systemic circulation (26). High progesterone concentration, and particularly an early rise of P_4_ during the first days after mating, is necessary to obtain larger embryo development, higher interferon-tau levels, and consequently lower embryo mortality—one of the main causes of reproductive failure in buffaloes (27–29). Moreover, it has already been demonstrated that an appropriate BFA around Day 10 after AI is required to ensure a proper CL function and to increase the likelihood of pregnancy in buffaloes (10).

During this time window (Day 5–10 post-AI), the first feature that changed (*P* < 0.05) between pregnant and not-pregnant animals was the P_4_ blood concentration at Day 7, followed by BFA at Day 8. The latter finding confirmed the previous result assessed by several studies performed in buffalo species (7, 30, 31). Because it has already been demonstrated that the use of B-mode ultrasonography alone to monitor CL features had some limits (32), the outcomes of the current study suggest an alternative approach to overcome this problem. Indeed, the evaluation of post-processing BFA by Doppler ultrasonography could be used, together with the P_4_ analysis, as a pregnancy predictor. Moreover, the development of the CLS analyzed by ANOVA repeated measure in both groups did not show any differences throughout the days, and BFA and P_4_ showed clear differences at the beginning and end of the trial. Another important result that arose from our study is that, for every analyzed feature, the transition from Day 9 to 10 seems to be a critical period for CL function and could be used to discriminate pregnant and not-pregnant animals. Further studies might be done to investigate the cellular and molecular mechanisms that cause this loss of BFA and CL functionality that started after Day 9 post-TAI in not-pregnant animals. It has already been found (7) that a higher expression of VEGF by luteal cells increases angiogenesis, vasculature development (reflected in increased Factor VIII), and BFA. It could be interesting to study whether the VEGF expression would change from Day 9 to 10 in both pregnant and not-pregnant animals.

To determine the performance of diagnostic tests, we used the ROC curve (33) to set a good cutoff value to predict pregnancy. Even if P_4_ blood levels at Day 5 could already give us some important information, more reliable results in terms of sensitivity and specificity could be recorded from Day 8. Moreover, to avoid a stressful condition for the animal, color Doppler ultrasonography and post-processing analyses, such as ImageJ, could also be used. In particular, we found that a BFA of 0.59 cm^2^ could be used as a predictor since Day 9 with good sensitivity (70.4%) and specificity (59.1%). However, our findings were in contrast to those of a study performed in cows (6) in which plasma P_4_ concentration had limited predictive power for pregnancy compared to BFA. The linear regression for BFA showed that, in not-pregnant animals, it was positively correlated with the P_4_ development during the last days (Δ5 = Day 10–9) and negatively correlated with the same parameter 2 days before (Δ3 = Day 8–7). This could be explained by the lower P_4_ levels produced by the CL during the Δ3 period. This means that, in not-pregnant animals, the CL is not well-developed, the luteinized phase has not already been completed, and it is difficult for the CL to grow and be vascularized to produce P_4_. The logistic regression analysis showed that pregnancy outcome prediction, from Day 5 to 10 after TAI, was positively influenced by P_4_ concentration and BFA.

It is worth pointing out that reproductive activity in buffalo species bred in the Mediterranean area is influenced by daylight length (3, 34) and that the breeding season corresponds to late autumn to early winter. Indeed, in sub-tropical zones and at higher latitudes, the reproductive function is mostly influenced by daylight length. During the breeding season, animals showed optimal ovarian function, higher P_4_ concentration, and a relatively higher conception rate (2, 30, 34). Our experiment was carried out during the breeding season in order to make our findings suitable at different latitudes (e.g., tropical and equatorial areas) where animals can show estrous cycles throughout the year, influenced mainly by nutrition. In the past, some studies (35, 36) reported that the use of BFA alone was not adequate for the early diagnosis of pregnancy in cattle due to low specificity and sensitivity and to a high variation between animals. However, in recent times, other studies (9, 37) have revealed that these limitations could be overcome because they are due to differences in Doppler ultrasound settings, in the criteria used for the statistical analysis, or in the times when BFA was measured post-AI. In cattle, most of the studies regarding BFA measurement started around Day 20 (32, 35, 37) because only between Day 17 and 21 did BFA decrease in not-pregnant animals due to an enhanced secretion of PGF2α (38). However, Kanazawa et al. (6) indicated that BFA evaluation on Day 7, measured retrospectively with ImageJ, could already be a reliable predictor of pregnancy in recipient cows subjected to embryo transfer. The same findings were reported in a study on bos indicus cattle (39) in which, starting from Day 7, BFA could discriminate pregnant animals from not-pregnant animals. Our findings are in agreement with the latter two studies (6, 39), suggesting that an early BFA measurement could be performed in both buffalo and different bovine species with good results in terms of pregnancy prediction. We are aware that postponing the time window of our analysis could give us better sensitivity and specificity values, but postponing the BFA evaluation also delays the resynchronization of open females. Moreover, there will always be animals that, due to asynchronized ovulations, fail to respond to the TAI or that undergo embryo mortality, giving us false positives.

In conclusion, the use of Doppler ultrasonography, together with a post-processing image tool, such as ImageJ, identified different patterns of BFA between pregnant and not-pregnant buffaloes starting from Day 8 post-TAI. The period between Day 9 and 10 is another important moment because, in those 24 h, there is a switch of all features (CLS, BFA, and P_4_) that significantly increase in pregnant animals while they decrease in the not-pregnant counterparts. Considering these findings, both P_4_ and BFA measurement seem to be better indexes of luteal function compared to CLS. Thus, BFA measurement at Day 9 or directly at Day 10 could be investigated to detect CL function and predict pregnancy. Moreover, molecular studies that underline the mechanisms of CL development and regression should be deeply studied to understand these phenomena. This will allow for an improvement in the productivity of herds with poor reproductive performance, particularly if early identification of luteolysis is associated with early resynchronization of not-pregnant females.

## Data Availability Statement

The original contributions presented in the study are included in the article/supplementary materials, further inquiries can be directed to the corresponding author/s.

## Ethics Statement

The animal study was reviewed and approved by The Ethical Animal Care and Use Committee of the Federico II University of Naples approved the experimental design and animal treatments. Written informed consent was obtained from the owners for the participation of their animals in this study.

## Author Contributions

AS and GC: conceptualization. MR and GF: methodology and data curation. GA and GZ: software. FL and GA: validation. AC and GZ: formal analysis. FL: investigation. GC: resources. AS and AC: writing—original draft preparation. GC and MR: writing—review & editing. GF: visualization. GC: supervision and funding acquisition. AS: project administration. All authors contributed to revision of the article for critical intellectual content and have approved the final version.

## Conflict of Interest

The authors declare that the research was conducted in the absence of any commercial or financial relationships that could be construed as a potential conflict of interest.
